# Expression of HER2/c-erbB-2, EGFR Protein in Gastric Carcinoma and its Clinical Significance

**DOI:** 10.1515/biol-2019-0013

**Published:** 2019-05-13

**Authors:** Guoxiong Cheng, Yijun Mei, Xiaoming Pan, Ming Liu, Suping Wu

**Affiliations:** 1 Department of Gastrointestinal surgery, Lishui Peoples’ Hospital of Zhejiang Province 323000 PR, Zhejiang China

**Keywords:** gastric cancer, HER2/c-erbB-2, EGFR, prognosis

## Abstract

**Objective:**

To investigate the HER2/c-erbB-2, epidermal growth factor receptor (EGFR) protein expression in gastric cancer and association with patients’ clinical pathology characteristics and prognosis.

**Methods:**

HER2/c-erbB-2 and EGFR protein expression was examined by immunohistochemical assay in gastric cancer tissue and corresponding paired normal gastric tissue of 67 patients of gastric carcinoma. The HER2/c-erbB-2, EGFR protein positive expression rate in cancer tissue and normal gastric tissue were compared. The correlation between HER2/c-erbB-2, EGFR protein positive expression and patients’ clinical pathology characteristics and survival was evaluated.

**Results:**

The positive expression rate of HER2/c-erbB-2 in the cancer and paired normal gastric tissues were 32.8% (22/67) and 4.5% (3/67), respectively with statistical difference (p<0.05). And the positive expression rate of EGFR in cancer and paired normal gastric tissues were 41.8% (28/67) and 5.9 (4/67), respectively, with statistical difference (p<0.05). HER2/c-erbB-2 positive expression in cancer tissue was significant correlated with the pathology grading (p<0.05), tumor invasion depth (p<0.05) and local regional lymph node metastasis (p<0.05); EGFR positive expression in cancer tissue was significant correlated with the tumor invasion depth (p<0.05) and local regional lymph node metastasis (p<0.05). The median survival time was 13.14 and 23.6 months respectively for HER2/c-erbB-2 positive and negative expression groups respectively with statistical difference ( HR=2.26, 9%CI:1.06-4.80, p<0.05). However, the median survival time was 15.47 and 22.87 months for EGFR positive and negative expression groups respectively, without statistical difference (HR=1.78, 9%CI:0.96-3.29, p>0.05).

**Conclusion:**

Positive expression of HER2/c-erbB-2 and EGFR proteins in cancer tissue was significant higher than normal gastric tissue and have significant correlation with prognosis.

## 1 Introduction

Gastric cancer is one of the most diagnosed malignant carcinomas in the digestive system. In 2012, more than 20,000 new cases of gastric cancer were diagnosed in the United States, with over 10,000 cases of deaths from gastric cancer being reported in the same year [1]. Although gastric cancer is one of the leading causes of cancer related death globally, the exact molecular mechanism of gastric cancer is not clear. As known, the over-expression of tumor oncogenes and the low expression of tumor suppressor genes may play important roles in the development of gastric cancer. HER2/c-erbB-2 is highly expressed in multiple malignancies of human beings and associated with the poor prognosis [2, 3]. The epidermal growth factor receptor (EGFR) gene is a proto-oncogene highly expressed in many tumors. Molecularly targeted drugs targeting EG FR have been used in the clinical treatment of various tumors[4]. In this study, we examined the HER2/c-erbB-2, EGFR expression in cancer and normal gastric tissue of 67 patients with gastric carcinomaby immunohistochemistry assay and explored the correlation between HER2/c-erbB-2, EGFR expression and clinicopathological features and prognosis.

## 2 Materials and methods

### 2.1 Patients

A total of 67 patients who suffered from gastric cancer and underwent general surgery from February 2012 to March 2016 in the People’s Hospital of Lishui, Zhejiang, China, were retrospectively analyzed. All patients had confirmed pathology diagnosis of gastric cancer and completed survival data. Gastric benign carcinoma and gastric cancer without enough survival data were excluded from the study. The immunohistochemical assay was performed to examine HER2/c-erbB-2 and EGFR protein expression in surgically resected cancerous tissues and paired normal gastric tissues in 67 patients with gastric cancer. The average age of the included 67 patients with gastric cancer was 62.3 ± 15.4 years (rang 43~79) with 41 males and 26 females. 32 patients were in I/II clinical stages, and other 35 were in the III/IV clinical stages. 17 cases were well/moderate differentiated adenocarcinomas, 50 cases were poor differentiated adenocarcinomas, 48 cases had regional lymph node metastases, and 19 cases without regional lymph node metastases.

**Informed consent**: Informed consent has been obtained from all individuals included in this study.

**Ethical approval**: The research related to human use has been complied with all the relevant national regulations, institutional policies and in accordance the tenets of the Helsinki Declaration, and has been approved by Medical Ethics Committee of Lishui Peoples’ Hospital of Zhejiang Province.

### 2.2 Immunohistochemical assay

Immunohistochemical assay was performed by the EnVision two-step method. The operation was carried out in accordance with laboratory procedures and instruction of the kit. Dewaxing and hydration were routinely performed, followed by 3% hydrogen peroxide application for 8 min to remove endogenous enzyme activity, citrate high-pressure antigen retrieval, and reagent A application at room temperature for 15 min. Primary antibody incubation was conducted overnight at 4 °C, and secondary antibody incubation was performed at 37 °C for 20 min. Reagent C incubation was implemented at 37 °C for 10 min. DAB was used for coloring and hematoxylin counterstaining.

### 2.3 Positive standards

The HER2/c-erbB-2, EGFR positive cells had mainly brownish yellow particles in the membrane and cytoplasm. The positive expression score of HER2/c-erbB-2 was follows: positive cells <5% was (−), 5%–25% was (+), 25%–50% was (++), and positive cells >50% was (+++).

For EGFR positive expression, the cells were mainly divided into 0 to 3 levels as follows: (i) Level 0, no brown-yellow particle-like precipitation observed in the cell membrane and the cytoplasm; (ii) level 1, the cell membrane and the cytoplasm showing a brown-yellow particle-like precipitation phenomenon with the percentage of positive cells was 10% or less recorded as “+”; (ii) level 2, cell membrane and cytoplasm exhibiting a brown-yellow particle-like precipitation phenomenon with the percentage of positive cells 10%–50%, recorded as “++;” (iv) level 3, the cell membrane and the cytoplasm displaying a brown-yellow particle-like precipitation phenomenon with the percentage of positive cells above 50% recorded as “+++.”

### 2.4 Statistical analysis

The statistical analysis was performed by STATA11.0 software. The measurement data were expressed with *x̄* ± *s* and the comparison between groups was made by student-t test of the sample mean. The enumeration data were expressed with number and compared by chi-square test. The survival data was expressed with the median and compared by log-rank test. P<0.05 meant a statistical difference.

## 3 Results

### 3.1 HER2/c-erbB-2 expression

**Figure 1:**
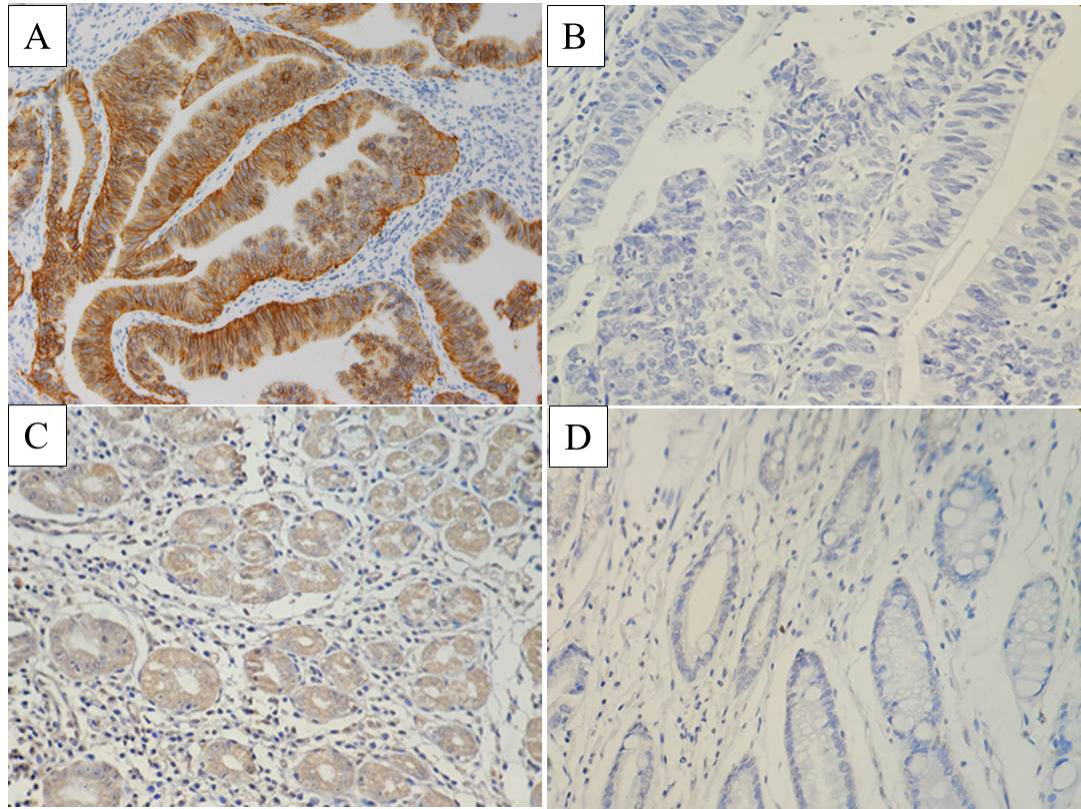
HER2/c-erbB-2 expression in gastric cancer and normal gastric tissue (A: HER2/c-erbB-2 positive expression in gastric cancer tissue; B: HER2/c-erbB-2 negative expression in gastric cancer tissue; C: HER2/c-erbB-2 positive expression in normal gastric tissue; D: HER2/c-erbB-2 negative expression in normal gastric tissue).

**Figure 2:**
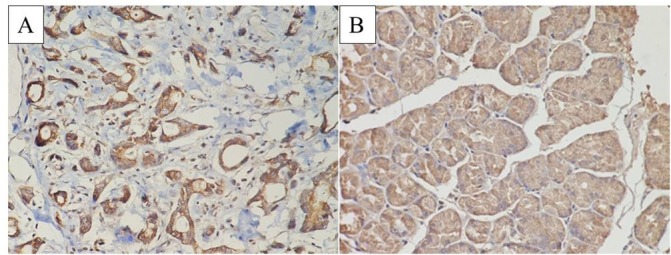
EGFR expression in gastric cancer and normal gastric tissue (A: EGFR positive expression in gastric cancer tissue; B: EGFR positive expression in normal gastric tissue).

HER2/c-erbB-2 protein mainly expressed in the cell membrane of gastric cancer cells with brownish yellow particles, (Figure 1). EGFR was mainly expressed in the membrane and cytoplasm of gastric cancer cells with uniform distribution of brown and yellow granules, (Figure 2). The positive rates of HER2/c-erbB-2 in the cancer and paired normal gastric tissues were 32.8% (22/67) and 4.5% (3/67), respectively with statistical difference (p<0.05). And the positive rates of EGFR in cancer and paired normal gastric tissues were 41.8% (28/67) and 5.9 (4/67), respectively with statistical difference (p<0.05). The HER2/c-erbB-2 and EGFR positive expression rate in the cancer tissues were significantly higher than that of normal gastric tissues.

### 3.2 Correlation between HER2/c-erbB-2expression and patients’ clinical features

HER2/c-erbB-2 positive expression in cancer tissue was significant correlated with the pathology grading (p<0.05), tumor invasion depth (p<0.05) and local regional lymph node metastasis (p<0.05); However, HER2/c-erbB-2 expression was not correlated with the patients age, gender, tumor location and tumor diameter (p>0.05), Table 1.

**Table 1:** Correlation between HER2/c-erbB-2 protein expression and patients’ clinical characteristics

Characteristics	n=67	HER2/c-erbB-2 expression		p-value
		Positive (n=22)	Negative (n=45)	
Gender				>0.05
Male	41	13	28	
Female	26	9	17	
Age(year)				>0.05
≤60	37	12	25	
＞60	30	10	20	
Tumor diameter(cm)				
≤5	24	8	16	
＞5	43	14	29	
Tumor location				>0.05
Near cardia	29	10	19	
Corpora ventriculi	8	2	6	
Near pylorus	30	10	20	
Pathology grading				<0.05
High differentiation	10	2	8	
Middle differentiation	7	1	6	
Low / undifferentiated	50	19	31	
Depth of invasion				<0.05
Invasion of serous layer	49	18	31	
Non invasion of serous layer	18	4	14	
Regional lymph node metastasis				<0.05
Positive	48	19	29	
Negative	19	3	16	

### 3.3 Correlation between EGFR expression and patients’ clinical features

EGFR positive expression in cancer tissue was significant correlated with the tumor invasion depth (p<0.05) and local regional lymph node metastasis (p<0.05), but was not correlated with the patients age, gender, tumor location, tumor diameter and pathology grading, (p>0.05), Table 2.

**Table 2:** Correlation between EGFR protein expression and patients’ clinical

Characteristics	n=67	EGFR expression		p-value
		Positive(n=28)	Negative(n=39)	
Gender				＞0.05
Male	41	18	23	
Female	26	10	16	
Age(year)				＞0.05
≤60	37	17	20	
＞60	30	11	19	
Tumor diameter(cm)				＞0.05
≤5	24	12	12	
＞5	43	16	27	
Tumor location				＞0.05
Near cardia	29	12	17	
Corpora ventriculi	8	3	5	
Near pylorus	30	13	17	
Pathology grading				＞0.05
High differentiation	10	4	6	
Middle differentiation	7	3	4	
Low / undifferentiated	50	21	29	
Depth of invasion				＜0.05
Invasion of serous layer	49	16	33	
Non invasion of serous layer	18	12	6	
Regional lymph node metastasis				＜0.05
Positive	48	14	34	
Negative	19	14	5	

### 3.4 HER2/c-erbB-2 expression and prognosis

**Figure 3:**
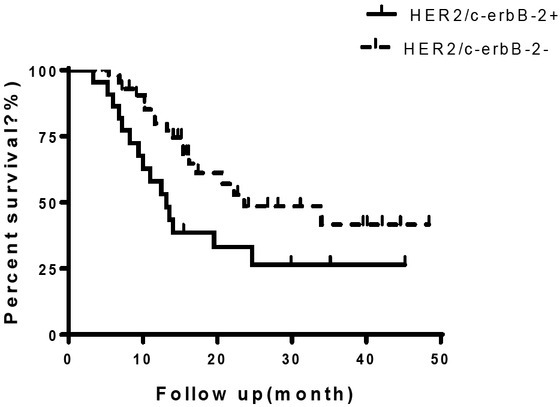
Survival curve of patients in HER2/c-erbB-2 positive and negative groups

The median survival time of were 13.14 and 23.6 months respectively for HER2/c-erbB-2 positive and negative expression groups respectively. The median survival time in positive group was significant lower than that of negative group with statistical difference (HR=2.26, 9%CI:1.06-4.80, p<0.05), Figure 3.

### 3.5 EGFR expression and prognosis

The median survival time of were 15.47 and 22.87 months respectively for EGFR positive and negative expression groups respectively without statistical difference (HR=1.78, 9%CI:0.96-3.29, p>0.05), Figure 2.

## 4 Discussion

The *HER2/c-erbB-2* gene is located in region 21 of chromosome 17 in human beings. The mRNA transcribed by HER2/c-erbB-2 encodes a 185 kD trans membrane protein with TK activity [5]. The expression level of

HER2/c-erbB-2 increases markedly in human malignant solid tumors, and this result is associated with the degree of the malignancy. Patients with a positive expression of HER2/c-erbB-2 often have a poorer prognosis with a shorter median survival time than those of patients with a negative expression [6, 7]. Yan et al. [8] performed immunohistochemistry assay to examin HER2/c-erbB-2 protein expression in 60 cases of advanced gastric cancer and found the positive expression rate of HER2/c-erbB-2 in gastric cancer is 36.7%. They also observed that HER2/c-erbB-2 expression is significantly correlated with tumor TNM stages and lymph node metastasis.

Our study showed the positive rate of HER2/c-erbB-2 in gastric cancer was 32.8%, which is slightly lower than that of Yan [8]. Our study also showed the positive expression of HER2/c-erbB-2 was related to the depth of tumor invasion (P < 0.05) and regional lymph node metastasis (P < 0.05). This finding was also consistent with previously published studies [5, 9, 10]. Therefore, we considered that HER2/c-erbB-2 might be related to the occurrence, development, invasion, and metastasis of gastric cancer. We also investigate the correlation between HER2/c-erbB-2 expression and patients prognosis. We found that the median survival times of HER2/c-erbB-2-positive and HER2/c-erbB-2-negative patients were 13.14 and 23.6 months, respectively, and the median survival time was significantly shorter in the patients with positive expression than that of patients with negative expression. The prognosis results of this study were also in accordance with the previously publications [11].

EGFR belongs to the tyrosine kinase type I receptor family, which mainly includes four types of homologous receptors: EGFR HER1, HER2, HER3, and HER4. The elevated EGFR expression can promote the proliferation, angiogenesis, adhesion, invasion, and metastasis of tumor cells in multiple solid tumors and inhibit the apoptosis of tumor cells. Studies have suggested that carcinomas with elevated EGFR expression had significantly enhanced malignant biological behavior, and was independent risk factor for poor prognosis [4, 12, 13]. Mizukami et al. [14] detected EGFR expression in patients with gastric cancer and a positive expression rate of 46% associated with tumor size, differentiation, and lymph node metastasis. In our study, EGFR-positive expression was significantly correlated with tumor invasion depth and regional lymph node metastasis (P < 0.05) but not with the age, gender, tumor diameter and pathology grading (P > 0.05). Some studies have also shown that the expression level of the EGFR protein elevated as the depth of invasion of tumors increases and the metastasis capacity enhancement [12, 15, 16].

Positive expression rates of HER2/c-erbB-2 and EGFR in gastric cancer tissue were significant higher than that of normal gastric tissue in patients with gastric cancer. This result suggested that HER2/c-erbB-2 and EGFR might be involved in biological behaviors, such as invasion and metastasis, of gastric cancer. HER2/c-erbB-2 and EGFR expression were related to the prognosis of gastric cancer patients and can be used as biomarkers of prognosis. However, the small sample size made the statistical power limited and larger sample size studies relevant to HER2/c-erbB-2, EGFR protein expression in gastric carcinoma and its clinical significance were needed to further discussion it correlation.

**Figure 4:**
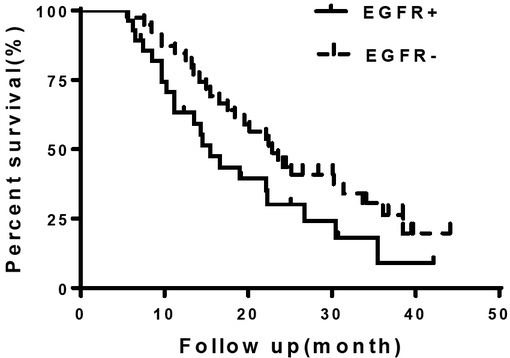
Survival curve of patients in EGFR positive and negative groups
